# Chronic treatment with valproate protects INS1 cell from palmitate-induced ER stress and apoptosis by inhibiting GSK3β

**DOI:** 10.1186/1687-9856-2013-S1-O21

**Published:** 2013-10-03

**Authors:** Shan Huang, Wei Wu, Yan Liang, Qin Ning, Xiao-ping Luo

**Affiliations:** 1 Department of Pediatrics, Tongji Hospital, Tongji Medical College, Huazhong University of Science and Technology, Wuhan, China; 2 Department of Infectious Diseases, Tongji Hospital, Tongji Medical College, Huazhong University of Science and Technology, Wuhan, China

## Objective

Reduction of β-cell mass is increasingly recognized as one of the main contributing factors to the pathogenesis of type 2 diabetes. Chronic free fatty acid (FFA) exposure has been shown to induce endoplasmic reticulum (ER) stress that may contribute to promoting pancreatic β-cell apoptosis. In the present study, we first investigated whether anticonvulsant sodium valproate (VPA), at clinically relevant doses, protects pancreatic β-cell from palmitate-induced apoptosis and the mechanism underlying anti-apoptosis.

## Methods and results

INS1 cells exposed to 0.25~1.0mM palmitate for 24~48h under serum-free conditions showed marked apoptosis in time- and concentration-dependent as assessed by CCK-8 assay, Hoechst 33342 / PI, flow cytometric cell apoptosis assay and electron microscopy. Palmitate triggered ER stress and apoptosis in INS1 cells as evidenced by increased mRNA levels of C/EBP homologous transcription factor (CHOP), activating transcription factor 4 (ATF4) and X box-binding protein 1 (XBP-1) in a time-dependent fashion. Western blot analysis also showed significant increase of CHOP and caspase-3 in protein level. We also found that palmitate activated GSK3β by inhibiting phosphorylation at serine 9. While chronic, not acute, 1~2mM VPA and 2mM LiCl remarkable reduced palmitate-induced cytotoxicity. Furthermore, INS1 cells treated with 10~20μM TDZD-8, a specific GSK3β inhibitor, also elicited cytoprotective responses against 0.25~0.5mM palmitate for 6~48h and decreased mRNA level of CHOP, but not ATF4 or XBP-1. The protein levels of CHOP, caspase-3 and GSK3β activity were remarkable reduced by co-treatment of INS1 cells with 0.25mM palmitate and 1mM VPA, compared with 0.25mM palmitate only. Finally, down-regulation of CHOP expression in INS1 cells by small interfering RNA (SiRNA) did not show apparent cytoprotective responses against 0.25mM palmitate.

## Conclusion

ER stress and GSK3β involved in palmitate-induced β-cell apoptosis, however, GSK3β other than ER stress is likely playing a more prominent role. Valproate protected pancreatic β-cell from palmitate-induced apoptosis and ER stress by inhibiting GSK3β.

